# Supplementary material for: Cross-Talk between Overlap Interactions in Biomolecules: A Case Study of the β-Turn Motif
Source: Molecules. 2021 Mar 11;26(6):1533. doi: 10.3390/molecules26061533 (PMC7998147; doi:10.3390/molecules26061533)
Supplement: Supplementary file 1 [file molecules-26-01533-s001.pdf]

SUPPLEMENTARY INFORMATION for

CROSS-TALK BETWEEN OVERLAP INTERACTIONS IN BIOMOLECULES: A CASE STUDY OF THE β-TURN MOTIF

Jayashree Nagesh†*

†Solid State and Structural Chemistry Unit, Indian Institute of Science Bangalore, Bengaluru 560012, Karnataka, India
*corresponding author jayashreen@iisc.ac.in

1) Table S1: Internal coordinate and backbone angle values for all the tetrapeptides used for characterizing the i+2 Ramachandran map and for proposing the geometric criteria. The coordinates d,λ are in Angstroms and θ, τ, ξ, Δ, φ and ψ are in degrees. The numbers in the interaction columns are stabilization energies from an NBO-Deletion analysis. The n-σ*CC is possible only when there is a Cβ, and for those with Glycine as the i+2 residue, the τ values are marked as NA (not applicable). The '-' means that the interaction energy is <0.01 kcal/mol or not present, since the algorithm screens interactions that are greater in strength than 0.01kcal/mol.

PDB (type)	Resi	d	θ	n-π*	τ	n-σ* CC	Ω	ξ	n-σ* CH	λ	Δ	π-π*	φ	ψ
3B1B (I)	214-217	3	113	0.38	162	0.47	2.7	52	-	3.7	25	-	-61	-29
1BZD (I)	60-63	3.1	106	0.09	158	0.56	2.2	41	0.02	3.7	15	-	-72	-30
1T8A(I)	20-23	3.2	137	-	155	0.49	2.5	35	0.53	4.2	36	-	-81	4
2BC3 (I)	115-118	3	114	0.35	163	0.50	2.7	50	-	3.7	22	0.08	-67	-29
3B1B (I)	147-150	3.6	136	-	136	0.26	2.5	15	2.1	4.6	46	-	-104	8
1K2A (I)	62-65	3.3	125	-	155	0.78	2.3	34	0.73	4.1	28	-	-89	-13
3SA0 (I)	22-25	3.3	115	-	153	0.40	2.7	29	0.44	3.5	22	0.07	-67	-36
1AYO (I')	75-78	3.2	119	0.19	75	-	2.3	8	0.34	4.0	-30	-	62	30
1D2I (I')	8-11	3	111	0.53	84	-	2.4	-14	0.35	3.6	-32	-	61	34
1AOK (I')	78-81	3.4	137	-	NA	-	3.9	-32	0.73	4.4	-47	-	84	-4
1D2O (I')	565-568	3.3	134	-	NA	-	4	-57	0.13	4.3	-45	-	83	1
1DXJ (I')	26-29	3.5	129	-	NA	-	3.8	-22	1.3	4.4	-33	-	94	2
1C0I (II)	1169-1172	2.9	119	0.76	NA	-	2.3	-9	2.1	3.7	-28	0.12	61	22
1A12(II)	396-399	3	115	0.46	81	-	2.4	-2	0.42	3.7	-27	-	63	26
1C0I (II)	1186-1189	3.7	141	-	NA	-	2.6	-53	0.67	4.7	-45	-	106	-14
1A1X (II)	73-76	3.3	107	0.07	73	-	3.8	19	0.25	3.8	-11	-	77	38
1B12 (II)	253-256	3.4	139	-	NA	-	3.8	-31	0.92	4.4	-50	-	86	-6

1AYE (II')	277-280	3.1	119	0.17	161	0.50	2.2	47	0	3.9	26	-	-71	-21
1DTD (II')	633-636	3.1	118	0.19	163	0.62	2.3	45	0.08	3.8	23	-	-69	-23
1C7T (II')	178-181	3.6	151	-	132	0.28	2.3	11.8	2.1	4.7	83	-	-109	30
1E8C (II')	415-418	3.4	135	-	147	0.43	2.5	27	0.88	4.4	47	-	-91	4
1H0H (II')	665-668	3.6	140	-	141	0.35	2.6	19.5	1.2	4.6	45	-	-99	10

2) Figure S1: Superposition of Ramachandran map in the main text on top of traditionally allowed regions of the Ramachandran map for any residue. The regions of various β-turns for the i+2 residue are also shown. It is seen that for regions away from the α-helical angles, interactions other than n-π* are favoured, such as n-σ^*_{CH} and n-σ^*_{CC}.

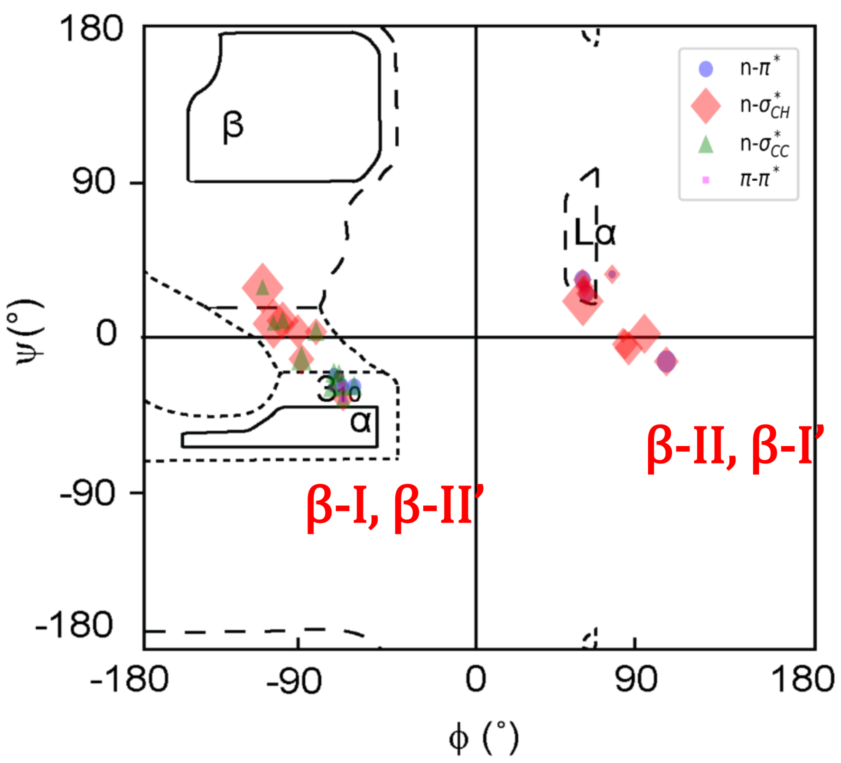

3) Coordinates of four tetrapeptides:

Hydrogen-optimised geometries of the four β-turn variant tetrapeptides

vA:

N	-27.36100000	23.57399900	5.73300000
C	-27.40000000	24.94500000	6.20400000
C	-28.57300000	25.23900000	7.13400000
O	-28.64699900	26.31300000	7.72300000
H	-27.47114900	25.60503000	5.33947800
H	-27.69902368	23.34949204	4.81903096
H	-26.48839400	25.11116600	6.77798800
N	-29.49399900	24.29100000	7.27300000

C	-30.76000000	24.56900000	7.94800000
C	-31.91400000	24.29000100	6.98200000
O	-32.19300100	23.13999900	6.64000000
C	-30.92499900	23.77199900	9.23500000
C	-29.72800100	23.96299900	10.14200000
C	-32.20800000	24.19300100	9.94700000
H	-32.45892300	25.21683500	9.66968900
H	-29.31665200	23.36636400	6.90735900
H	-30.76467500	25.61953400	8.23861900
H	-30.99196200	22.71365200	8.98297400
H	-29.37593800	22.99165700	10.48934500
H	-32.06198100	24.13348600	11.02553400
H	-30.01400400	24.57388300	10.99822600
H	-33.02050400	23.52823400	9.65370500
H	-28.93096500	24.46176900	9.59058100
N	-32.58499900	25.35100000	6.55200000
C	-33.59199900	25.24000000	5.50500000
C	-33.01499900	24.67200100	4.19800000
O	-33.65700100	23.86000100	3.56200000
C	-34.25000000	26.60199900	5.24800000
H	-34.42955000	26.72364200	4.17979400
H	-32.39345900	26.25512900	6.95938600
H	-34.34614600	24.53690900	5.85859300
H	-33.59137300	27.39638500	5.59909200
H	-35.19804400	26.65365200	5.78338900
N	-31.80900000	25.07700000	3.80000000
C	-31.22100100	24.60500000	2.54700000
C	-30.29100000	23.42499900	2.81800000
O	-29.39301841	23.51996384	3.69445953
C	-30.46400100	25.72900000	1.82800000
C	-31.29500000	26.98200000	1.52200000
C	-30.47300000	28.01400000	0.77300000
C	-32.56800100	26.62500000	0.73500000
H	-32.92338900	25.64206900	1.04426500
H	-31.29002200	25.72394800	4.37638600
H	-32.03015500	24.27866200	1.89364600
H	-30.08798000	25.33375000	0.88434500
H	-31.59761600	27.41745200	2.47431600
H	-30.33420400	28.89412500	1.40086600
H	-32.34471500	26.61216400	-0.33180800
H	-29.67534600	26.04747200	2.50968800
H	-30.99214700	28.29816400	-0.14233400
H	-33.33895500	27.36904300	0.93532900
H	-29.50052600	27.59048500	0.52194300
H	-26.99606354	22.85310250	6.32217731
H	-30.40273308	22.51722650	2.26269396

vS:

H	-27.500000	25.497999	5.476000
H	-27.847000	23.405001	5.052000
H	-26.691000	25.047001	7.048000

H	-26.541000	23.173000	6.034000
H	-33.450001	28.257000	5.729000
H	-32.335999	26.350000	7.009000
H	-34.266998	24.528999	5.930000
H	-34.889000	26.840000	6.397000
H	-35.216999	26.398001	4.706000
H	-30.819000	27.750000	-0.307000
H	-31.197001	25.674999	4.858000
H	-31.665001	24.757000	2.050000
H	-30.166000	22.834999	2.307000
H	-29.167000	25.662001	2.002000
H	-31.521999	27.510000	2.020000
H	-29.393000	28.516001	3.141000
H	-30.850000	26.011000	0.073000
H	-29.834999	26.499001	3.539000
H	-30.160999	29.410000	1.807000
H	-29.299000	26.882999	0.022000
H	-28.695999	28.445999	1.505000
H	-32.609001	24.753000	10.233000
H	-29.377001	23.408001	7.236000
H	-31.010000	25.552999	8.623000
H	-30.884001	22.594000	9.021000
H	-29.462999	22.815001	10.717000
H	-32.293999	23.212000	11.067000
H	-30.135000	24.379999	11.234000
H	-33.097000	23.216999	9.479000
H	-28.992001	24.308001	9.871000
N	-27.495001	23.504000	5.994000
C	-27.540001	24.893999	6.382000
C	-28.773001	25.312000	7.172000
O	-28.924000	26.462000	7.493000
N	-29.622000	24.353001	7.494000
C	-30.896999	24.555000	8.199000
C	-31.993000	24.336000	7.141000
O	-32.249001	23.205000	6.714000
C	-30.978001	23.610001	9.404000
C	-29.808001	23.791000	10.377000
C	-32.342999	23.705000	10.096000
N	-32.585999	25.438999	6.651000
C	-33.585999	25.316000	5.607000
C	-32.971001	24.884001	4.292000
O	-33.665001	24.254999	3.474000
C	-34.433998	26.579000	5.442000
O	-33.605000	27.650999	5.001000
N	-31.687000	25.207001	4.109000
C	-30.945000	24.910000	2.854000
C	-30.094999	23.660000	3.016000
O	-29.320999	23.610001	3.978000
C	-30.013000	26.077000	2.550000
C	-30.488001	27.284000	1.759000
C	-29.622999	28.500999	2.076000

C	-30.355000	26.959000	0.283000

vT:

H	8.691000	-12.473000	-2.758000
H	10.738000	-13.091000	-3.210000
H	9.577000	-11.857000	-1.289000
H	11.228000	-13.181000	-1.637000
H	3.691000	-19.198999	-3.775000
H	5.621000	-15.947000	-1.211000
H	5.900000	-18.573000	-2.228000
H	4.259000	-16.263000	-3.334000
H	3.390000	-16.733000	-1.384000
H	4.746000	-18.288000	-4.881000
H	3.053000	-17.789000	-4.653000
H	5.060000	-16.872999	-7.320000
H	6.740000	-15.403000	-3.682000
H	7.653000	-16.829000	-6.115000
H	9.977000	-16.299000	-5.786000
H	7.751000	-14.568000	-7.125000
H	5.086000	-15.194000	-5.809000
H	4.960000	-13.694000	-8.273000
H	6.616000	-16.341000	-8.004000
H	7.162000	-13.917000	-5.527000
H	5.873000	-12.806000	-7.029000
H	5.093000	-15.646000	-8.609000
H	4.216000	-13.361000	-6.691000
H	9.338000	-15.742000	3.107000
H	9.597000	-15.116000	-1.109000
H	7.004000	-15.252000	0.430000
H	9.505000	-16.892000	0.764000
H	7.621000	-17.089001	2.936000
H	8.327000	-14.543000	2.264000
H	8.280000	-18.375999	1.897000
H	10.007000	-14.815000	1.743000
H	6.810000	-17.500999	1.406000
N	10.483000	-13.401000	-2.283000
C	9.261000	-12.727000	-1.864000
C	8.359000	-13.536000	-0.944000
O	7.371000	-13.018000	-0.402000
N	8.712000	-14.796000	-0.743000
C	7.872000	-15.742000	-0.012000
C	8.636000	-16.375000	1.170000
C	7.775000	-17.410000	1.906000
C	9.111000	-15.291000	2.141000
C	7.462000	-16.790001	-1.036000
O	8.242000	-17.688000	-1.376000
N	6.246000	-16.663000	-1.554000
C	5.818000	-17.556999	-2.615000
C	6.716000	-17.423000	-3.848000
O	7.126000	-18.431999	-4.430000
C	4.353000	-17.299999	-3.011000

O	3.506000	-17.546000	-1.881000
C	3.931000	-18.209000	-4.162000
N	7.021000	-16.188999	-4.251000
C	7.750000	-15.941000	-5.491000
C	9.241000	-15.716000	-5.232000
O	9.621000	-14.881000	-4.402000
C	7.151000	-14.747000	-6.233000
C	5.695000	-14.912000	-6.668000
C	5.148000	-13.601000	-7.203000
C	5.610000	-16.024000	-7.727000

vV:

H	-16.93000000	-17.82900000	59.32500000
H	-15.36300000	-19.59000000	59.07300000
H	-15.46600000	-17.21400000	60.20500000
H	-15.34000000	-19.80200000	60.81000000
H	-14.87000000	-19.19500000	63.58700000
H	-15.77400000	-19.10600000	62.39400000
H	-17.98900000	-17.67500000	63.80800000
H	-16.06700000	-17.43700000	65.05300000
H	-17.10200000	-18.01100000	66.89400000
H	-15.35900000	-20.35500000	64.84500000
H	-16.47300000	-19.66600000	66.70900000
H	-14.32400000	-18.97000000	65.26500000
H	-18.05800000	-19.23500000	66.02500000
H	-22.74400000	-19.61700000	62.15800000
H	-19.90100000	-18.61500000	63.48000000
H	-20.14100000	-21.42900000	64.24300000
H	-22.56300000	-21.09800000	64.03000000
H	-21.07300000	-20.23600000	66.02100000
H	-21.79500000	-18.35700000	62.98300000
H	-22.81000000	-19.85400000	65.96100000
H	-23.42500000	-18.76900000	63.56700000
H	-21.61300000	-18.63700000	65.45900000
H	-20.64600000	-20.35600000	57.85700000
H	-19.48600000	-19.76700000	61.39700000
H	-20.70600000	-21.88500000	59.70200000
H	-18.50100000	-22.40000000	58.70600000
H	-19.61700000	-19.21100000	58.75300000
H	-21.31200000	-19.45400000	59.23900000
N	-15.58300000	-19.25100000	59.99900000
C	-16.24900000	-17.97100000	60.16400000
C	-17.04400000	-17.84500000	61.45000000
O	-18.02500000	-17.12500000	61.52400000
N	-16.61800000	-18.55700000	62.47900000
C	-17.35300000	-18.55700000	63.73100000
C	-18.27000000	-19.78300000	63.76700000
O	-17.81300000	-20.91200000	63.90900000
C	-16.37800000	-18.47400000	64.92500000
C	-17.05000000	-18.87500000	66.23100000
C	-15.14700000	-19.30700000	64.63500000

N	-19.56700000	-19.56200000	63.59200000
C	-20.51100000	-20.66400000	63.56000000
C	-20.60100000	-21.26800000	62.15700000
O	-21.24100000	-22.30200000	61.97600000
C	-21.91400000	-20.22300000	64.04900000
C	-21.84800000	-19.70100000	65.47300000
C	-22.51200000	-19.16600000	63.12300000
N	-19.98400000	-20.61600000	61.17100000
C	-19.99100000	-21.06600000	59.78300000
C	-18.60200000	-21.55100000	59.38200000
O	-17.59200000	-20.99300000	59.81500000
C	-20.42200000	-19.94100000	58.84000000

4) Figure S2: GB1 protein showing Loop L1 β-turn. The i+1-i+2 'KT' segment is engineered to 'vV','vS','vA' and 'vT' where 'v' refers to D-valine.

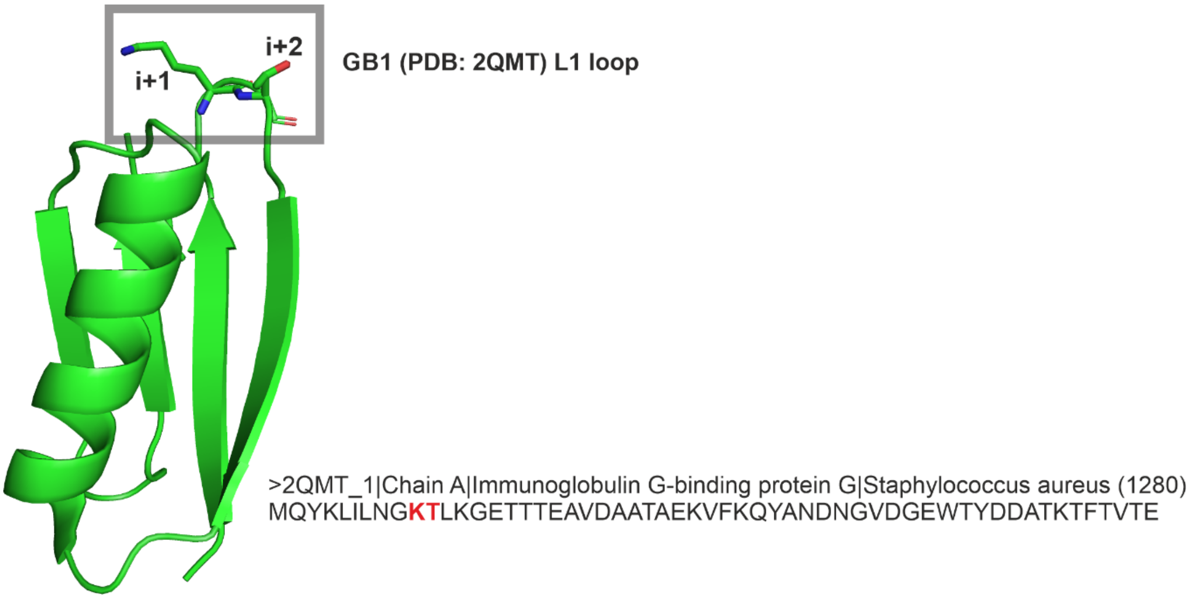

5) Table S2: Amino acid sequences of the 22 proteins used in this study.

PDB (type)	Index	Residues in β-turn	Sequence
3B1B (I)	6	214-217	>3B1B_1|Chains A,B|Carbonic anhydrase 1|Chlamydomonas reinhardtii (3055)<br>MARTGALLLVALALAGCAQACIYKFGTSPDSKATVSGDHWDHGLNGENWEGKDGAGNAWVCKT<br>GRKQSPINVPQYQVLDGKGSKIANGLQTQWSYPDLMSNGTSVQVINNGHTIQVQWTYNYAGHA<br>TIAIPAMHNQTNRIVDVLEMRPNDAADRVTAVPTQFHFHSTSEHLLAGKIYPLELHIVHQVTE<br>KLEACKGGCFSVTGILFQLDNGPDNELLEPIFANMPSREGTFSNLPAGTTIKLGELLPSDRDY<br>VTYEGSLTTPPCSEGLLWHVMTQPQRISFGQWNRYRLAVGLKECNSTETAADAGHHHHHRRLL<br>HNHAHLEEVPAATSEPKHYFRRVMLAESANPDAYTCKAVAFGQNFRNPQYANGRTIKLARYH
1BZD (I)	5	60-63	>1BZD_1|Chains A,B|PROTEIN (TRANSTHYRETIN)|Homo sapiens (9606)<br>GPTGTSESKCPLMVKVLDAVRGSPAINVAVHVFRKAADDTWEPFASGKTSESGELHGLTTEEE<br>FVEGIYKVEIDTKSYWKALGISPFHEHAEVVFTANDSGPRRYTIAALLSPYSYSTTAVVTNPK<br>E
1T8A(I)	3	20-23	>1T8A_1|Chain A|Lysozyme|Enterobacteria phage T4 (10665)<br>MNIFEMLRIDEGLRLKIYKDTEGYYTIGIGHLLTKSPSINAAKSELDKAINAAKSELDKAIGA<br>NTNGVITKDEAEKLFNQDVDAAVRGILRNAKLKPVYDSLDAVRRAALINMVFQMGETGVAGFT<br>NSLRMLQQKRWDEAAVNLAKSRWYNQTPNRAKRVITTFRTGTWDAYKNL

<table>
<tr><td>2BC3
(I)</td><td>7</td><td>115-
118</td><td>>2BC3_1|Chains A,B|Streptavidin|Streptomyces avidinii (1895)
MASMTGGQQMGRDEAGITGTWYNQLGSTFIVTAGADGALTGTYESAVGNAESRYVLTGRYDSA
PATDGSGTALGWTVAWKNNYRNAHSATTWSGQYVGGAEARINTQWLLTSGTTEANAWKSTLVG
HDTFTKVKPSAASIDAAKKAGVNNGNPLDAVQQ</td></tr>
<tr><td>3B1B
(I)</td><td>1</td><td>147-
150</td><td>>3B1B_1|Chains A,B|Carbonic anhydrase 1|Chlamydomonas reinhardtii (3055)
MARTGALLLVALALAGCAQACIYKFGTSPDSKATVSGDHWDHGLNGENWEGKDGAGNAWVCKT
GRKQSPINVPQYQVLDGKGSKIANGLQTQWSYPDLMSNGTSVQVINNGHTIQVQWTYNYAGHA
TIAIPAMHNQTNRIVDVLEMRPNDAADRVTAVPTQFHFHSTSEHLLAGKIYPLELHIVHQVTE
KLEACKGGCFSVTGILFQLDNGPDNELLEPIFANMPSREGTFSNLPAGTTIKLGELLPSDRDY
VTYEGSLTTPPCSEGLLWHVMTQPQRISFGQWNRYRLAVGLKECNSTETAADAGHHHHHRRLL
HNHAHLEEVPAATSEPKHYFRRVMLAESANPDAYTCKAVAFGQNFRNPQYANGRTIKLARYH</td></tr>
<tr><td>1K2A
(I)</td><td>2</td><td>62-65</td><td>>1K2A_1|Chain A|eosinophil-derived neurotoxin|Homo sapiens (9606)
HVKPPQFTWAQWFETQHINMTSQQCTNAMQVINNYQRRCKNQNTFLLTTFANVVNVCGNPNMT
CPSNKTRKNCHHSGSQVPLIHCNLTTPSPQNISNCRYAQTPANMFYIVACDNRDQRRDPPQYP
VVPVHLDRII</td></tr>
<tr><td>3SA0
(I)</td><td>4</td><td>22-25</td><td>>3SA0_1|Chain A|Mitogen-activated protein kinase 1|Homo sapiens (9606)
MAAAAAAGAGPEMVRGQVFDVGPRYTNLSYIGEGAYGMVCSAYDNVNKVRVAIKKISPFEHQT
YCQRTLREIKILLRFRHENIIGINDIIRAPTIEQMKDVYIVQDLMETDLYKLLKTQHLSNDHI
CYFLYQILRGLKYIHSANVLHRDLKPSNLLLNTTCDLKICDFGLARVADPDHDHTGFLTEYVA
TRWYRAPEIMLNSKGYTKSIDIWSVGCILAEMLSNRPIFPGKHYLDQLNHILGILGSPSQEDL
NCIINLKARNYLLSLPHKNKVPWNRLFPNADSKALDLLDKMLTFNPHKRIEVEQALAHPYLEQ
YYDPSDEPIAEAPFKFDMELDDLPKEKLKELIFEETARFQPGYRS</td></tr>
<tr><td>1AYO
(I’)</td><td>11</td><td>75-78</td><td>>1AYO_1|Chains A,B|ALPHA-2-MACROGLOBULIN|Bos taurus (9913)
EFPFALEVQTLPQTCDGPKAHTSFQISLSVSYIGSRPASNMAIVDVKMVSGFIPLKPTVKMLE
RSNVSRTEVSNNHVLIYLDKVTNETLTLTFTVLQDIPVRDLKPAIVKVYDYYETDEFAVAEYS
APCSSNN</td></tr>
<tr><td>1D2I
(I’)</td><td>10</td><td>8-11</td><td>>1D2I_1|Chains C,D|DNA (5'-D(*TP*AP*TP*TP*AP*TP*AP*GP*AP*TP*CP*TP*AP*TP*AP*A)-3')|null
TATTATAGATCTATAA
>1D2I_2|Chains A,B|PROTEIN (RESTRICTION ENDONUCLEASE BGLII)|Bacillus subtilis (1423)
MKIDITDYNHADEILNPQLWKEIEETLLKMPLHVKASDQASKVGSLIFDPVGTNQYIKDELVP
KHWKNNIPIPKRFDFLGTDIDFGKRDTLVEVQFSNYPFLLNNTVRSELFHKSNMDIDEEGMKV
AIIITKGHMFPASNSSLYYEQAQNQLNSLAEYNVFDVPIRLVGLIEDFETDIDIVSTTYADKR
YSRTITKRDTVKGKVIDTNTPNTRRRKRGTIVTY</td></tr>
<tr><td>1AOK
(I’)</td><td>9</td><td>78-81</td><td>>1AOK_2|Chain B|VIPOXIN COMPLEX|Vipera ammodytes meridionalis (73841)
NLFQFAKMINGKLGAFSVWNYISYGCYCGWGGQGTPKDATDRCCFVHDCCYGRVRGCNPKLAI
YYYSFKKGNIVCGKNNGCLRDICECDRVAANCFHQNKNTYNANYKFLSSSRCRQTGEKC</td></tr>
<tr><td>1D2O
(I’)</td><td>12</td><td>565-
568</td><td>>1D2O_1|Chains A,B|COLLAGEN ADHESIN|Staphylococcus aureus (1280)
ETTSSIGEKVWDDKDNQDGKRPEKVSVNLLANGEKVKTLDVTSETNWKYEFKDLPKYDEGKKI
EYTVTEDHVKDYTTDINGTTITNKYTPGETSATVTKNWDDNNNQDGKRPTEIKVELYQDGKAT
GKTAILNESNNWTHTWTGLDEKAKGQQVKYTVEELTKVKGYTTHVDNNDMGNLITTNKYTP</td></tr>
<tr><td>1DXJ
(I’)</td><td>8</td><td>26-29</td><td>>1DXJ_1|Chain A|CLASS II CHITINASE|CANAVALIA ENSIFORMIS (3823)
DVGSVIDASLFDQLLKHRNDPACEGKGFYSYNAFVTAARSFGGFGTTGDTNTRKREVAAFLAQ
TSHETTGGAAGSPDGPYAWGYCFVTERDKSNKYCDPGTPCPAGKSYYGRGPIQLTHNYNYAQA
GRALGVDLINNPDLVARDAVISFKTAIWFWMTPQGNKPSCHDVITNRWTPSAADVAANRTPGF
GVITNIINGGIECGRGPSPASGDRIGFYKRYCDVLHLSYGPNLNCRDQRPFGG</td></tr>
<tr><td>1C0I
(II)</td><td>15</td><td>1169-
1172</td><td>>1C0I_1|Chain A|D-AMINO ACID OXIDASE|Rhodosporidium toruloides (5286)
LMMHSQKRVVVLGSGVIGLSSALILARKGYSVHILARDLPEDVSSQTFASPWAGANWTPFMTL
TDGPRQAKWEESTFKKWVELVPTGHAMWLKGTRRFAQNEDGLLGHWYKDITPNYRPLPSSECP
PGAIGVTYDTLSVHAPKYCQYLARELQKLGATFERRTVTSLEQAFDGADLVVNATGLGAKSIA
GIDDQAAEPIRGQTVLVKSPCKRCTMDSSDPASPAYIIPRPGGEVICGGTYGVGDWDLSVNPE
TVQRILKHCLRLDPTISSDGTIEGIEVLRHNVGLRPARRGGPRVEAERIVLPLDRTKSPLSLG
RGSARAAKEKEVTLVHAYGFSSAGYQQSWGAAEDVAQLVDEAFQRYHG</td></tr>
<tr><td>1A12(II
)</td><td>16</td><td>396-
399</td><td>>1A12_1|Chains A,B,C|REGULATOR OF CHROMOSOME CONDENSATION 1|Homo sapiens (9606)
RRSPPADAIPKSKKVKVSHRSHSTEPGLVLTLGQGDVGQLGLGENVMERKKPALVSIPEDVVQ
AEAGGMHTVCLSKSGQVYSFGCNDEGALGRDTSVEGSEMVPGKVELQEKVVQVSAGDSHTAAL
TDDGRVFLWGSFRDNNGVIGLLEPMKKSMVPVQVQLDVPVVKVASGNDHLVMLTADGDLYTLG
CGEQGQLGRVPELFANRGGRQGLERLLVPKCVMLKSRGSRGHVRFQDAFCGAYFTFAISHEGH</td></tr>
</table>

			VYGFGLSNYHQLGTPGTESCFIPQNLTSFKNSTKSWVGFSGGQHHTVCMDSEGKAYSLGRAEY<br>GRLGLGEGAEEKSIPTLISRLPAVSSVACGASVGYAVTKDGRVFAWGMGTNYQLGTGQDEDAW<br>SPVEMMGKQLENRVVLSVSSGGQHTVLLVKDKEQS
1C0I (II)	13	1186-1189	>1C0I_1|Chain A|D-AMINO ACID OXIDASE|Rhodosporidium toruloides (5286)<br>LMMHSQKRVVVLGSGVIGLSSALILARKGYSVHILARDLPEDVSSQTFASPWAGANWTPFMTL<br>TDGPRQAKWEESTFKKWVELVPTGHAMWLKGTRRFAQNEDGLLGHWYKDITPNYRPLPSSECP<br>PGAIGVTYDTLSVHAPKYCQYLARELQKLGATFERRTVTSLEQAFDGADLVVNATGLGAKSIA<br>GIDDQAAEPIRGQTVLVKSPCKRCTMDSSDPASPAYIIPRPGGEVICGGTYGVGDWDLSVNPE<br>TVQRILKHCLRLDPTISSDGTIEGIEVLRHNVGLRPARRGGPRVEAERIVLPLDRTKSPLSLG<br>RGSARAAKEKEVTLVHAYGFSSAGYQQSWGAAEDVAQLVDEAFQRYHG
1A1X (II)	17	73-76	>1A1X_1|Chain A|HMTCP-1|Homo sapiens (9606)<br>GSAGEDVGAPPDHLWVHQEGIYRDEYQRTWVAVVEEETSFLRARVQQIQVPLGDAARPSHLLT<br>SQLPLMWQLYPEERYMDNNSRLWQIQHHLMVRGVQELLLKLLPDD
1B12 (II)	14	253-256	>1B12_1|Chains A,B,C,D|SIGNAL PEPTIDASE I|Escherichia coli (469008)<br>VRSFIYEPFQIPSGSMMPTLLIGDFILVEKFAYGIKDPIYQKTLIETGHPKRGDIVVFKYPED<br>PKLDYIKRAVGLPGDKVTYDPVSKELTIQPGCSSGQACENALPVTYSNVEPSDFVQTFSRRNG<br>GEATSGFFEVPKNETKENGIRLSERKETLGDVTHRILTVPIAQDQVGMYYQQPGQQLATWIVP<br>PGQYFMMGDNRDNSADSRYWGFVPEANLVGRATAIWMSFDKQEGEWPTGLRLSRIGGIH
1AYE(II’)	22	277-280	>1AYE_1|Chain A|PROCARBOXYPEPTIDASE A2|Homo sapiens (9606)<br>LETFVGDQVLEIVPSNEEQIKNLLQLEAQEHLQLDFWKSPTTPGETAHVRVPFVNVQAVKVFL<br>ESQGIAYSIMIEDVQVLLDKENEEMLFNRRRERSGNFNFGAYHTLEEISQEMDNLVAEHPGLV<br>SKVNIGSSFENRPMNVLKFSTGGDKPAIWLDAGIHAREWVTQATALWTANKIVSDYGKDPSIT<br>SILDALDIFLLPVTNPDGYVFSQTKNRMWRKTRSKVSGSLCVGVDPNRNWDAGFGGPGASSNP<br>CSDSYHGPSANSEVEVKSIVDFIKSHGKVKAFIILHSYSQLLMFPYGYKCTKLDDFDELSEVA<br>QKAAQSLRSLHGTKYKVGPICSVIYQASGGSIDWSYDYGIKYSFAFELRDTGRYGFLLPARQI<br>LPTAEETWLGLKAIMEHVRDHPY
1DTD (II’)	21	633-636	>1DTD_1|Chain A|CARBOXYPEPTIDASE A2|Homo sapiens (9606)<br>FNFGAYHTLEEISQEMDNLVAEHPGLVSKVNIGSSFENRPMNVLKFSTGGDKPAIWLDAGIHA<br>REWVTQATALWTANKIVSDYGKDPSITSILDALDIFLLPVTNPDGYVFSQTKNRMWRKTRSKV<br>SAGSLCVGVDPNRNWDAGFGGPGASSNPCSDSYHGPSANSEVEVKSIVDFIKSHGKVKAFIIL<br>HSYSQLLMFPYGYKCTKLDDFDELSEVAQKAAQSLSRLHGTKYKVGPICSVIYQASGGSIDWS<br>YDYGIKYSFAFELRDTGRYGFLLPARQILPTAEETWLGLKAIMEHVRDHPY
1C7T (II’)	18	178-181	>1C7T_1|Chain A|BETA-N-ACETYLHEXOSAMINIDASE|Serratia marcescens (615)<br>DQQLVDQLSQLKLNVKMLDNRAGENGVDCAALGADWASCNRVLFTLSNDGQAIDGKDWVIYFH<br>SPRQTLRVDNDQFKIAHLTGDLYKLEPTAKFSGFPAGKAVEIPVVAEYWQLFRNDFLPRWYAT<br>SGDAKPKMLANTDTENLDQFVAPFTGDQWKRTKDDKNILMTPASRFVSNADLQTLPAGALRGK<br>IVPTPMQVKVHAQDADLRKGVALDLSTLVKPAADVVSQRFALLGVPVQTNGYPIKTDIQPGKF<br>KGAMAVSGAYELKIGKKEAQVIGFDQAGVFYGLQSILSLVPSDGSGKIATLDASDAPRFPYRG<br>IFLDVARNFHKKDAVLRLLDQMAAYKLNKFHFHLSDDEGWRIEIPGLPELTEVGGQRCHDLSE<br>TTCLLPQYGQGPDVYGGFFSRQDYIDIIKYAQARQIEVIPEIDMPAHARAAVVSMEARYKKLH<br>AAGKEQEANEFRLVDQTDTSNTTSVQFFNRQSYLNPCLDSSQRFVDKVIGEIAQMHKEAGQPI<br>KTWHFGGDDAKNIRLGAGYTDKAKPEPGKGIIDQSNEDKPWAKSQVCQTMIKEGKVADMEHLP<br>SYFGQEVSKLVKAHGIDRMQAWQDGLKDAESSKAFATSRVGVNFWDTLYWGGFDSVNDWANKG<br>YEVVVSNPDYVYMDFPYEVNPDERGYYWGTRFSDERKVFSFAPDNMPQNAETSVDRDGNHFNA<br>KSDKPWPGAYGLSAQLWSETQRTDPQMEYMIFPRALSVAERSWHRAGWEQDYRAGREYKGGET<br>HFVDTQALEKDWLRFANILGQRELAKLDKGGVAYRLPVPGARVAGGKLEANIALPGLGIEYST<br>DGGKQWQRYDAKAKPAVSGEVQVRSVSPDGKRYSRAEKV
1E8C (II’)	20	415-418	>1E8C_1|Chains A,B|UDP-N-ACETYLMURAMOYLALANYL-D-GLUTAMATE--2,6-DIAMINOPIMELATE LIGASE|ESCHERICHIA COLI (562)<br>ADRNLRDLLAPWVPDAPSRALREMTLDSRVAAAGDLFVAVVGHQADGRRYIPQAIAQGVAAII<br>AEAKDEATDGEIREMHGVPVIYLSQLNERLSALAGRFYHEPSDNLRLVGVTGTNGKTTTTQLL<br>AQWSQLLGEISAVMGTVGNGLLGKVIPTENTTGSAVDVQHELAGLVDQGATFCAMEVSSHGLV<br>QHRVAALKFAASVFTNLSRDHLDYHGDMEHYEAAKWLLYSEHHCGQAIINADDEVGRRWLAKL<br>PDAVAVSMEDHINPNCHGRWLKATEVNYHDSGATIRFSSSWGDGEIESHLMGAFNVSNLLLAL<br>ATLLALGYPLADLLKTAARLQPVCGRMEVFTAPGKPTVVVDYAHTPDALEKALQAARLHCAGK<br>LWCVFGCGGDRDKGKRPLMGAIAEEFADVAVVTDDNPRTEEPRAIINDILAGMLDAGHAKVME<br>GRAEAVTCAVMQAKENDVVLVAGKGHEDYQIVGNQRLDYSDRVTVARLLGVIARSHH
1H0H (II’)	19	665-668	>1H0H_1|Chains A,K|FORMATE DEHYDROGENASE SUBUNIT ALPHA|DESULFOVIBRIO GIGAS (879)<br>ATMALKTVDAKQTTSVCCYCSVGCGLIVHTDKKTNRAINVEGDPDHPINEGSLCAKGASTWQL<br>AENERRPANPLYRAPGSDQWEEKSWDWMLDTIAERVAKTREATFVTKNAKGQVVNRCDGIASV<br>GSAAMDNEECWIYQAWLRSLGLFYIEHQARIUHSATVAALAESYGRGAMTNHWIDLKNSDVIL<br>MMGSNPAENHPISFKWVMRAKDKGATLIHVDPRYTRTSTKCDLYAPLRSGSDIAFLNGMTKYI<br>LEKELYFKDYVVNYTNASFIVGEGFAFEEGLFAGYNKETRKYDKSKWGFERDENGNPKRDETL

			KHPRCVFQIMKKHYERYDLDKISAICGTPKELILKVYDAYCATGKPDKAGTIMYAMGWTQHTV<br>GVQNIRAMSINQLLLGNIGVAGGGVNALRGEANVQGSTDHGLLMHIYPGYLGTARASIPTYEE<br>YTKKFTPVSKDPQSANWWSNFPKYSASYIKSMWPDADLNEAYGYLPKGEDGKDYSWLTLFDDM<br>FQGKIKGFFAWGQNPACSGANSNKTREALTKLDWMVNVNIFDNETGSFWRGPDMDPKKIKTEV<br>FFLPCAVAIEKEGSISNSGRWMQWRYVGPEPRKNAIPDGDLIVELAKRVQKLLAKTPGKLAAP<br>VTKLKTDYWVNDHGHFDPHKIAKLINGFALKDFKVGDVEYKAGQQIATFGHLQADGSTTSGCW<br>IYTGSYTEKGNMAARRDKTQTDMQAKIGLYPGWTWAWPVNRRIIYNRASVDLNGKPYAPEKAV<br>VEWNAAEKKWVGDVPDGPWPPQADKEKGKRAFIMKPEGYAYLYGPGREDGPLPEYYEPMECPV<br>IEHPFSKTLHNPTALHFATEEKAVCDPRYPFICSTYRVTEHWQTGLMTRNTPWLLEAEPQMFC<br>EMSEELATLRGIKNGDKVILESVRGKLWAKAIITKRIKPFAIQGQQVHMVGIPWHYGWSFPKN<br>GGDAANILTPSVGNPNTGIPETKAFMVNVTKA
